# Assessment of Drivers with Alzheimer’s Disease in High Demand Driving Situations: Coping with Intersections in a Driving Simulator

**DOI:** 10.3390/geriatrics1030021

**Published:** 2016-08-31

**Authors:** Arne Stinchcombe, Stephanie Paquet, Stephanie Yamin, Sylvain Gagnon

**Affiliations:** 1 School of Psychology, University of Ottawa, Ottawa, ON K1N 6N5, Canada; astinchc@uottawa.ca (A.S.); stephaniepaquet230@gmail.com (S.P.); 2 Faculty of Human Sciences, Saint Paul University, Ottawa, ON K1S 1C4, Canada; syamin@ustpaul.ca

**Keywords:** driving, dementia, Alzheimer’s disease, simulation, intersections, attention, driver assessment

## Abstract

Intersections are one of the most complex and cognitively demanding driving situations. Individuals with dementia and, more precisely, Alzheimer’s disease (AD), may face additional challenges negotiating intersections given the nature of their cognitive decline, which often includes deficits of attention. We developed a comprehensive evaluation scheme to assess simulated driving performance at intersections. The evaluation scheme captured all types of errors that could occur during preparation (i.e., prior to the intersection), execution (i.e., during the intersection), and recovery (i.e., after the intersection). Using the evaluation scheme, intersection behaviour in a driving simulator among 17 drivers with mild AD was compared to that of 21 healthy controls. The results indicated that across all types of intersections, mild AD drivers exhibited a greater number of errors relative to controls. Drivers with mild AD made the most errors during the preparation period leading up to the intersection. These findings present a novel approach to analyzing intersection behaviour and contribute to the growing body of research on dementia and driving.

## 1. Introduction

The complexity of driving combined with its ubiquity and consideration as an instrumental activity of daily living [1] makes the cognitive integrity of the driver a safety priority [2]. It is known that at-fault crashes are most often caused by driving errors, some of which are cognitive in nature [3,4,5]. While driving errors in younger drivers have been associated to risk-taking, among older drivers findings indicate that the risk of crash is associated with health-related cognitive and functional decline [6]. A challenge faced by transportation authorities and health professionals consists of determining the fitness to drive for patients with neurological disorders, whose driving will ultimately be compromised due to their illness.

The most common form of age-associated cognitive impairment is dementia, a term used to describe persistent cognitive impairment in several domains (e.g., memory, attention, language, executive function, and visuospatial abilities) as well as functional impairment. The most prevalent form of dementia is Alzheimer’s disease (AD). While drivers in the mild stage of dementia may continue to drive safely, all drivers with dementia will, at some point, have to cease driving as their disease progresses and cognitive declines affect their ability to drive safely [2]. In many jurisdictions, physicians are required to evaluate AD patients’ safety and suitability to drive and make a recommendation regarding driving fitness [7,8]. Considering the importance of driving for a significant majority of older drivers, one of the challenges involves determining when in the course of disease one’s safety risk is significantly inflated.

Previous studies have used tests of global cognition (e.g., Mini-Mental State Exam; MMSE) to predict driving outcomes among older drivers [9,10]. It is commonly accepted that higher scores on these tests are associated with better driving performance. Within practice settings, healthcare professionals may use a combination of tests of global cognition, on-road exams, and reports from families to make a decision regarding fitness to drive [7,11,12]. However, there is no single cognitive test or domain that yields sufficient specificity and sensitivity indices to discriminate safe drivers from unsafe older drivers [13,14]. Indeed, our research has recently demonstrated that among drivers with AD, results on measures of attention were more strongly associated with driving performance in the simulator in comparison to a test of general cognition [15] and we suggested that attention deficits are perhaps a stronger predictor of driving ability in this population. However, if limitations in attention are one of the primary underlying causes of driving difficulties in AD drivers, errors in attention should also be more frequently observed in AD when assessed on the road.

Attention is a critical component of safe driving and attentional deficits can be present in AD [16]. Given the importance of attention to driving, one would expect that drivers with AD would have greater difficulty than healthy older drivers of the same age executing the various maneuvers that are necessary to safely drive. For example, attentional failures have been associated with errors occurring at intersections, especially when executing a left turn [17]. In a recent on-road study, Aksan and colleagues [18] compared the driving performance of healthy older drivers to the drivers with AD and drivers with Parkinson’s Disease (PD). Their participants were tested in a baseline condition and a navigation condition. In the baseline condition, participants had to drive according to the instructions received. In the navigation condition participants had to find their way based on their memory of a short route or were asked to name restaurants and road signs and a segment of similar length. Their results showed that AD drivers did not commit more errors at intersections while tested in the baseline condition. Interestingly, in the navigation condition, there was an increase in errors in both the AD and healthy group, with AD patients making significantly more errors. The authors attributed the navigation effect to a dual-task effect similar to divided attention on driving performance [19]. However, it is still unknown whether deficits in attention are a major determinant of driving difficulties among AD drivers and whether they impair the ability of AD drivers to execute the appropriate behaviours at intersections.

We examined the nature of driving errors in a sample of mild AD drivers who were tested in a driving simulator [15]. In our previous published analysis of our data, we concluded that mild AD drivers displayed significantly more driving errors overall compared to healthy controls. One specific type of driving error at intersections and was recorded by the simulator was when a driver did not stop at an intersection (red/yellow light or all-way stop). Although the number of errors made by AD participants was significantly greater than errors made by control participants (i.e., a mean of 2 among mild AD drivers vs. 43 for controls), this type of error only amounted to approximately 5% of the total number of errors found in the mild AD group. It is possible that many more errors occur at intersections that were not accounted for by simulator scores. For example, we observed more crashes overall in the group of AD drivers but could not document whether they occurred at intersections or not.

In the present study, we investigate driving errors at intersections made by mild AD drivers and neurologically healthy drivers using the playback function of the driving simulator. Using this function, we were able to replay the entire scenario from the driver’s perspective or from a topographical “bird’s eye” view. We developed a systematic scoring procedure to assess performance at intersections. This scoring procedure was partly based on the conceptual framework of Stanton and Salmon [20] and Vlahodimitrakou and colleagues [21] who developed on-road assessment at intersections that we adapted to the simulator context. Unless additional equipment is installed, simulators do not allow video capture of the driver. Therefore, the scoring system that we developed allows scoring performance based on the playback function of the simulator. Our goal was to capture all types of errors that could occur during preparation (i.e., prior the intersection), execution (i.e., during the intersection), and recovery (i.e., after the intersection) as well as to document whether these errors are more frequently observed among mild AD drivers.

## 2. Materials and Methods

The present study consists of a targeted analysis of behaviour at intersections. The data for this study were collected as part of a broader investigation, which is detailed in Yamin, Stinchcombe, and Gagnon [15].

### 2.1. Participants

Thirty-eight drivers participated in this study. A group of 17 participants (10 men and 7 women) had a diagnosis of mild AD. The control group was made up of 21 neurologically normal participants (10 men, 11 women). All participants held a valid driver’s licence at the time of the study. They all gave informed consent prior to the study. This study received approval from the Research Ethics Boards (REB) of University of Ottawa as well as Bruyère Continuing Care in Ottawa, Ontario (Canada).

The participants in the AD group were recruited at a local memory clinic. Based on review of patient files, patients at the clinic who had received an AD diagnosis, and were in the early stages of the illness were contacted to determine their interest in participating in the study. All participants received an AD diagnosis from a neurologist by applying the diagnostic criteria established by the National Institute of Neurological and Communicative Diseases and stroke and the Association of Alzheimer’s and related diseases (NINCDS-ADRDA). The global deterioration scale (GDS) was administered in order to include only participants in the early stages of AD (stage 3 and 4) [22]. Potential participants with substance abuse problems, learning difficulties, or suffering from severe visual (ex. cataracts), auditory, or other health problems, possibly having an influence on their performance, such as mental illness, a history of head trauma, heart attack, epilepsy, apoplexy, hypertension, or sleep apnoea were not selected for the study. At the time of the study, all but one participant from the AD group were taking an acetylcholinesterase inhibitor. APOE4 carrier status was not available and thus is not presented.

Participants in the control group consisted of a convenience sample recruited through an announcement published in a community newspaper. Participants in the control group were age matched to individuals in the AD group. A score less than 25 in the Mini Mental State Exam (MMSE) [23], the use of medication altering cognitive functions, or any other condition previously mentioned and documented for its potential effects on cognition represented the exclusion criteria for the control group. Scores on the MMSE of less than 25 may indicate cognitive impairment [24]. None of the participants in the control group had a score of less than 25 on the MMSE.

### 2.2. Driving Simulator

The simulator was installed in a room of a local memory disorders clinic. It consisted of a Dell Precision M6300 laptop (Intel Core 2 DUO processor, 2.10 GHz/2.0 GB RAM), with a 17-inch screen, operating under the Windows XP operating system and equipped with a steering wheel and a command console on the floor including an accelerator and a brake pedal (Logitech, model G25). The route simulation and recording of data was programmed using the STISIM simulation software (Version 2.08.004) designed by the Systems Tech (Hawthorne CA, USA). The field of view of the simulation from this software was 60 degrees horizontally and 75 degrees vertically. The frame rate was 30 frames/s 30 Hz). For the needs of this study, two route courses were used. The first course was designed with the goal of familiarizing the participants with the simulator. The second scenario, of a significantly longer length, was developed in order to evaluate their driving skills based on existing standards of a provincial licensing authority in Canada. Prerecorded verbal commands, transmitted through the computer speakers, dictated the required maneuvers for both courses to the participants, such as a lane change, or which direction to go at the different intersections. Other realistic noises, such as acceleration, slowing down, the use of brakes causing the wheels to lock, or auditory indications of a crash could be heard.

The execution of the first course (i.e., the familiarization course) was preceded by in depth explanations of the required task, the functioning of the simulator, and the devices used to control the virtual vehicle (e.g., brakes, wheel, rotating the view to see the blind spots, accelerator). The course familiarizing the participant with the simulator, completed by all participants, was 6.6 km in length. It was used to practice speed control, turn execution, familiarization with signs, and practicing turns at intersections. The scenario simulated residential routes in an urban area.

The second route completed by the participants (i.e., experimental course), which was 12.3 km in length, mimicked a route in the region of Thunder Bay, Ontario (Canada), including residential, and urban sectors, highway segments, and 16 controlled intersections. Twelve intersections were controlled by traffic lights, and four others were signaled by stop signs. The test course that was studied has been used in several other studies [15,19,25] and has been shown to result in important similarities between performance on the simulated route and performance on real roads [25]. The test course lasted approximately 20 min.

**Figure 1:**
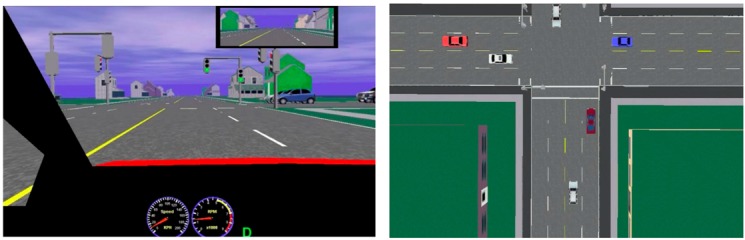
Viewing angles used to evaluate intersections. Driver’s view (**left**) and bird’s eye view (**right**).

The simulator allows for a video playback of each participant’s drive. This video offers two viewing angles: one in the position of the driver, and the other is a bird’s eye view from above the driver’s vehicle (see Figure 1). It is from these two angles of analysis of the recording that we identified the driving errors of each participant, and then classified them. Two observers previously trained to use the evaluation scheme independently rated each participants’ drive. The observers were blind to which group participants belonged.

### 2.3. Driving Errors Evaluation Scheme

A driving error evaluation scheme was constructed based on taxonomies developed by Stanton and Salmon [20] and by Vlahodimitrakou et al. [21]. These taxonomies allow for the discrimination of errors according to their underlying nature (attentional, perceptual, judgment, or coordination). A thorough review of these taxonomies, the intersections themselves, and other possible errors at intersections were taken into consideration. By considering the driving evaluation criteria developed in these two studies, and other types of errors identified from a comprehensive analysis of road behaviours at intersections, the probable errors at an intersection were identified and classified according to the probability of their location of occurrence (before, during, or after the intersection) (see Appendix Table A1).

The period before the intersection was identified as the region spanning from the verbal command to the first intersection line. An example of an error that could occur before the intersection could include exceeding the speed limit. The period during the intersection was identified as the area between the two intersection lines of two different adjacent roads in the intersection, and the period after the intersection was identified as the following 30 m after the second intersection line. An example of an error that could occur during the intersection is very slow execution while turning. Some errors occurred before and throughout the intersection, preventing ongoing analysis of intersection errors. These errors, named “errors of initiation”, were classified using two subcategories: “errors of omission”, where the requested behaviour asked of the participant was simply not executed at the intersection, and “incapacity of execution”, where the position of the vehicle impeded the execution of the requested maneuver at the intersection (See Table 1). Errors that occurred after the intersection were considered “recovery errors”. An example of a recovery error is loss of control of the vehicle following the intersection.

Another added error category, “crash”, prevented the driver from completing the requested maneuver during the intersection. In fact, during a crash the driver’s simulated windshield shatters, regardless of the nature of the crash or the force of the impact. Following a short interval, the driver could take control of the vehicle at the region of the crash.

Once the evaluation scheme was completed, two trained raters watched the recorded videos of the 38 participants. The data collected in the present study resulted from having two independent raters code intersections and apply the above-mentioned evaluation scheme.

### 2.4. Procedure

The procedures for this study are detailed elsewhere [15] and only a brief summary is presented here. To begin, each participant in the control group completed a telephone interview approximately 20 min in length, to verify their eligibility for the study, as well as a brief demographic questionnaire. Following that, the mild AD participants were contacted by phone to verify their interest, and also completed a demographic questionnaire if they wanted to participate. Each participant took part in two test sessions at a local memory disorders clinic, at weekly intervals, each approximately 2 h and 30 min in length. The first session consisted of the administration of a variety of neuropsychological tests [see 15]. During the second testing session, the participants completed two simulated driving scenarios: the first was the familiarization phase and the second was test phase. Data from the first session (i.e., neuropsychological data) were not considered in the analyses pertaining to this study.

### 2.5. Statistical Analyses

Two blind raters independently reviewed each participant’s drive and documented errors that occurred at the 16 controlled intersections according to the driving errors evaluation scheme. To determine inter-rater reliability, Pearson Correlation coefficients were computed between raters. In general, the correlations were high with the highest correlation reaching r = 0.96 between raters. Variables for which correlations were below r = 0.70 were excluded from further analysis. Six variables were excluded for this reason. Additionally, none of the participants made errors that were characterized as ‘unrequested turn executed’ or ‘excessive braking’ following the intersection. Thus, these error types are not included in the analysis. For each type of error, an average between the two raters was computed and used as the dependent variable in the analysis.

In total, 26 variables were included in the analysis. Data were analyzed through independent samples *t*-tests treating group (i.e., mild AD vs. control) as the between variable.

## 3. Results

The results are presented according to preparation errors, execution errors, and recovery errors. Table 2, Table 3 and Table 4 present a summary of the results.

### 3.1. Participant Characteristics

The average age of participants in the AD group was 79.5 years old (SD = 6.8, range from 67 to 90 years). Participants in the AD group had a mean MMSE score of 25.06 (SD = 3.83, range from 13 to 30) and a mean GDS score of 3.05 (SD = 0.24, range from 3 to 4).

The average age of participants in the control group was 77 (SD = 5.9, range from 68 to 86 years). Participants in the control group had a mean MMSE score of 29.00 (SD = 1.3, range from 25 to 30) and a mean GDS score of 1.19 (SD = 0.4, range from 1 to 2).

All participants completed the simulated drive and none of the participants reported any discomfort associated with the driving simulator, a condition often referred to as s simulator adaptation syndrome.

### 3.2. Preparation Errors

In total, mild AD drivers exhibited an average of 67.68 errors in the preparation period prior to the intersection in comparison to 33.60 errors among controls. The results showed that during this period, AD drivers were more likely to avoid using their brakes [t (36) = 4.48, *p* < 0.001)], and were also more likely to use their brakes inappropriately [t (36) = 3.27, *p* = 0.002] and stop inappropriately after the verbal command [t (36) = 2.29, *p* = 0.028]. AD drivers also drove faster than control drivers [t (36) = 3.91, *p* < 0.001] and were more likely to be in the wrong lane [t (36) = 2.60, *p* = 0.013)] or on the shoulder leading up to the intersection [t (36) = 2.879, *p* = 0.007)]. In comparison to controls, AD drivers were more likely to disobey the signal light and make an action that did not correspond with the traffic light [t (36) = 2.157, *p* = 0.038)]. No differences were observed in terms of following too closely. The results showing differences in preparation errors are presented in Table 1.

### 3.3. Execution Errors

The average number of errors committed during the intersection was 8.41 among AD drivers and 5.62 among controls. In terms of vehicle control errors during the execution of intersections, AD drivers had difficulties controlling the vehicle (i.e., loss of control of the vehicle) in comparison to controls [t (36) = 4.29, *p* < 0.001]. No differences were observed in terms of speed of execution and excessive stopping. There were also no statistically significant differences between groups observed during left turns. In terms of traffic light errors during the intersection, AD drivers were also more likely to disobey the traffic light signal and enter the intersection inappropriately [t (36) = 2.46, *p* < 0.019]. The results showing differences in errors that occurred during the intersection appear in Table 2.

### 3.4. Recovery Errors

During the recovery period, AD drivers committed an average of 7.82 errors after the intersection compared to an average of 5.00 committed by controls. Of all of the error types that were examined in the recovery period, only one variable was deemed significant. Indeed, following the intersection, the results showed that AD drivers were more likely to lose control of the vehicle in comparison to controls [t (36) = 2.39, *p* = 0.022]. No statistically significant differences were observed in terms of speed, stopping following the intersection, or incorrect orientation following the intersection. The results are presented in Table 3.

### 3.5. Initiation Errors and Crashes

As detailed in the method section, in several instances due to more grievous errors, intersections could not be rated using the error types noted above. Overall, AD drivers made an average of 5.59 initiation errors compared to 1.40 among controls. AD drivers were more likely to not respect the auditory instructions provided by the simulator [t (36) = 4.02, *p* < 0.001] and were found not to execute the requested turn [t (36) = 4.28, *p* < 0.001]. In comparison to controls, AD drivers were also more likely to drive off of the road [t (36) = 2.36, *p* = 0.023]. There were no statistically significant differences between groups in terms of opposite turns that were executed. In a final analysis, we examined the number of crashes in the two groups at intersection. The data indicate that AD drivers were over three times as likely to crash in and around an intersection, a difference that reached statistical significance [t (36) = 3.69, *p* = 0.001]. Results related to initiation errors and to crash can be found in Table 4.

## 4. Discussion

This study compared errors at intersections among mild AD drivers and healthy control drivers. We developed a systematic evaluation scheme to assess performance at intersections. The goal of this study was to capture all types of errors that could occur at intersections and to document whether these errors are more frequently observed among AD drivers. In order to do this, we separated the intersections into three periods: preparation, execution, and recovery.

Our findings showed that across all types of intersections, AD drivers exhibited a greater number of errors relative to healthy controls. In particular, in the preparation period AD drivers were more likely to drive at higher speeds, use their brakes inappropriately, and enter the intersection at an inappropriate time. In contrast, AD drivers seemed to have less difficulty executing the behaviours once at the intersection as well as in the recovery period as evidenced by a smaller number of errors in these sections. Nevertheless, similar to the preparation phase, we noted more vehicle control errors (e.g., losing control of the vehicle) in the AD group while only a few instances of these errors were noticed in healthy controls. Another type of error that was found to be more frequent in the AD group is associated with the decisions at traffic lights. We observed that AD drivers tended to disobey the traffic light signal (e.g., not stopping on an amber light) more frequently than the control participants. In the recovery period, errors more frequently observed in the AD group were exclusively associated with vehicle control.

Our analysis of the playback of the simulated drives also allowed us to confirm that AD drivers crashed in the simulator more frequently on average than did the healthy controls at intersections. We had evidence in our previous study that the occurrence of crash in the simulator was greater in AD drivers as recorded by the simulator during the entire drive [15]. Here, we confirmed that crashes in the simulator occurred almost exclusively at intersections. This finding clearly indicates that the errors observed in the AD group are severe enough to lead to crashes in the simulator. On average, AD drivers crashed 3 times at intersections in a scenario that contained only 16 intersections. Crashes in the simulator were infrequent in healthy older individuals mostly because they are very strategic while driving in a simulator. This is evidenced by the fact that healthy older drivers compensated by driving at lower speed in the preparation period. It is important to note that these crashes occurred in the simulator environment and are not necessarily indicative of real-world crashes. Unlike simulated driving, where other road users are not responsive to the driver, during real-world driving conditions other road users play an active role by reacting to unsafe driving maneuvers to avoid crash.

Moreover, in the present study as well as in our previous analysis of AD driving performance [15], AD drivers were found to drive too fast and used the brake inappropriately on many occasions. These results shed light on the underlying mechanisms that lead to driving errors among individuals with AD. On the one hand, it could be argued that AD drivers could not adapt to the simulated driving context and thus could not control the vehicle. In this instance, their errors would be primarily due to difficulties in handling the vehicle. If this were the case, however, crashes would have been observed throughout the scenario yet, when contrasting these results with our previous work, we observe that crashes primarily occurred at intersections. On the other hand, given that AD drivers crashed at intersections where visual complexity tends to be high, a more plausible conclusion is that errors resulted from deficits in attention and judgement. Disobeying the traffic light more frequently is also another indication, that AD drivers, on average, had difficulty coping with the attentional demands at intersections. Inappropriate use of brakes is an indication that AD drivers had issues with judgement suggesting that some of their errors may also stem from deficits in executive function.

These findings are in line with existing literature which shows that AD drivers have more difficulties negotiating intersections relative to controls [15,26]. In a recent investigation of on-road safety among drivers with neurodegenerative diseases (i.e., AD and PD) and controls, researchers sought to examine safety errors when completing a secondary task [18]. Their results showed that in the absence of a secondary task, drivers with neurodegenerative diseases exhibited more errors overall and showed more lane observance errors. When a secondary task was added, drivers with neurodegenerative diseases showed difficulties turning and observing their lane as well as more errors overall. Our findings differ somewhat from the results cited above, insofar as we noted striking differences in intersection behaviour between AD drivers and healthy controls on a driving simulator when no external secondary task had to be performed while driving. In our study, it is plausible that, due to the novelty of using a driving simulator, the simulated driving task increases workload in a similar fashion as a secondary task. Along the same line, the simulated route is novel and participants could not expect where they would be asked to go. As such, they have to wait for the directional instructions before making any decision prior to the intersections. It is likely that this also increased the attentional demands at intersections.

A main difference between our results and previous findings on this issue comes from our detailed analysis of the intersections in a segmented manner. As others, we documented errors in the execution of the intersection per se in AD drivers. However, the preparation period seems to be the most challenging segment for our AD drivers and illustrates the challenges faced by AD drivers in these situations. The approach presented here offers a sensitive assessment of intersection errors over and above the errors documented automatically by the driving simulator (exclusively while at the execution stage). The evaluation grid allows for a moment-by-moment analysis of the nature and location of errors made prior to, during, and immediately following intersections.

Therefore, our findings highlight the relevance of a systematic approach to analyzing intersections that is highly sensitive to differences in driving behaviour. The results show that this approach can reliably differentiate between AD drivers and healthy drivers. The evaluation scheme presented here is advantageous in that it captures errors that occur in the preparatory period prior to an intersection and our findings suggest that this is a particularly challenging period for AD drivers.

While these findings contribute to the literature on driving and cognitive impairment, the study is not without limitations. In particular, the raters’ assessment of intersection behaviour was based on two perspectives: the driver’s view and a bird’s eye view. Research suggests that visual scanning behaviours are an important factor in safe driving [27] and, in our study, we were unable to examine scanning behaviours as there was no camera facing the drivers. We speculate that many of the errors we observed (e.g., not obeying traffic light), especially those that lead to crashes, may be related to attention. Scanning behaviours are a direct reflection of allocation of attention and these could be observed with additional cameras facing the driver. Future research would benefit from examining differences in scanning behaviours at intersections among AD drivers. Indeed, the addition of scanning behaviours could definitely improve the evaluation framework presented here. Finally, this study did not include an on-road component which would allow for the comparison of these findings relative to naturalistic driving. Indeed, it is possible that some of our findings may be attributed to the novelty of the driving simulator which could be assessed through a comparison with on-road driving. Such a comparison would also demonstrate the generalizability of the findings to the real world.

Many drivers in the mild stage of dementia can drive safely [2]. However, it is important for clinicians and researchers to consider cognitive factors as well as performance during complex roadway situations in making a formal recommendation regarding driving fitness. Evidence from this study suggests that the preparatory period should be scrutinized when examining fitness to drive among AD drivers. This paper adds to the growing body of research on dementia and driving and presents a novel approach to analyzing intersection behaviour.

## Figures and Tables

**Table 1:** Summary of preparation errors.

Errors	Mean AD	SD AD	Mean Control	SD Control	*t*	*df*	*p*
**Brakes and speed**							
No use of brakes	1.500	1.146	0.262	0.490	4.482	36	<0.001
Braking	8.824	7.462	3.000	3.021	3.269	36	0.002
Exceeding speed limit by 5 km/h to 15 km /h	10.529	4.605	5.143	3.870	3.919	36	<0.001
Exceeding speed limit by more than 15 km/h	7.500	4.822	2.000	2.225	4.661	36	<0.001
**Inappropriate stop**							
Excessive preparation	6.912	6.129	5.714	5.083	0.659	36	0.514
Frequency of inappropriate stops after the verbal command	11.176	8.107	6.548	4.022	2.296	36	0.028
Exceeding the stop line	0.500	0.685	0.429	0.482	0.377	36	0.708
**Choice of lane**							
Lane deviation	14.059	8.624	7.405	7.144	2.603	36	0.013
Wrong lane	1.735	1.786	0.524	1.054	2.602	36	0.013
Shoulder lane	0.441	0.704	0.000	0.000	2.879	36	0.007
**Signaling**							
Disobeying signal	4.147	2.720	2.357	2.393	2.157	36	0.038
**Safety**							
Follows too closely	0.353	0.702	0.190	0.512	0.825	36	0.415
Right of way not given to pedestrian	0.000	0.000	0.024	0.109	−0.897	36	0.375

Mean refers to average number of errors while SD stands for standard deviation; AD are Alzheimer’s disease patients and Control refer to the neurologically healthy group; *t* stands for the *t*-test value of the pair comparison, *df* for degrees of freedom and *p* for the probability of the obtained *t* value.

**Table 2:** Summary of execution errors.

Errors	Mean AD	SD AD	Mean Control	SD Control	*t*	*df*	*p*
**Vehicle control**							
Excessive stopping and anticipation for a right turn	0.324	0.611	0.857	1.415	0.019	36	0.157
Very slow execution	1.706	2.100	2.310	2.052	−0.892	36	0.378
Incapable of controlling the vehicle	3.324	2.639	0.643	1.002	4.298	36	<0.001
**Left turn**							
Vehicle is an obstruction in the intersection.	0.471	0.624	0.238	0.407	1.384	36	0.175
Insufficient time to complete turn	0.059	0.166	0.000	0.000	1.629	36	0.112
Missed turn	0.118	0.281	0.238	0.375	−1.097	36	0.280
**Traffic light**							
Disobeying signal	2.412	1.593	1.333	1.099	2.464	36	0.019

Mean refers to average number of errors while SD stands for standard deviation; AD are Alzheimer’s disease patients and Control refer to the neurologically healthy group; *t* stands for the *t*-test value of the pair comparison, *df* for degrees of freedom and *p* for the probability of the obtained *t* value.

**Table 3:** Summary of recovery errors.

Errors	Mean AD	SD AD	Mean Control	SD Control	*t*	*df*	*p*
**Speed**							
Speed remains constant	1.118	0.781	0.810	0.661	1.317	36	0.196
**Stopping**							
Unnecessary stop after the intersection	0.206	0.532	0.143	0.359	0.435	36	0.666
**Vehicle orientation**							
Wrong orientation	4.353	2.377	3.262	1.758	1.626	36	0.113
Loss of control	2.147	2.269	0.786	1.168	2.391	36	0.022

Mean refers to average number of errors while SD stands for standard deviation; AD are Alzheimer’s disease patients and Control refer to the neurologically healthy group; *t* stands for the *t*-test value of the pair comparison, *df* for degrees of freedom and *p* for the probability of the obtained *t* value.

**Table 4:** Summary of initiation errors and occurrence of crash.

Errors	Mean AD	SD AD	Mean Control	SD Control	*t*	*df*	*p*
**Initiation errors**							
**Omission**	1.588	1.121	0.357	0.761	4.022	36	<0.001
Requested turn not executed	1.529	1.125	0.286	0.644	4.283	36	<0.001
Opposite turn executed	0.059	0.243	0.071	0.239	−0.161	36	0.873
**Vehicle control**	1.735	2.768	0.095	0.201	2.716	36	0.010
Driving in the opposite direction	0.765	1.276	0.048	0.150	2.561	36	0.015
Driving on the shoulder	0.147	0.386	0.048	0.150	1.086	36	0.285
Driving off the road	0.824	1.600	0.000	0.000	2.366	36	0.023
**Crash**							
Frequency of crash	3.265	2.641	0.952	1.036	3.687	36	0.001

Mean refers to average number of errors while SD stands for standard deviation; AD are Alzheimer’s disease patients and Control refer to the neurologically healthy group; *t* stands for the *t*-test value of the pair comparison, *df* for degrees of freedom and *p* for the probability of the obtained *t* value.

## Appendix

**Table A1:** Evaluation Scheme—Error Names and Definitions.

Errors	Definition	Nature
**Initiation errors**	**All errors prevent adequate analysis of the intersection.**	
**Omission**	**Does not respect the verbal command.**	
Requested turn not executed	The participant continues straight, omitting the requested turn	Judgement
Unrequested turn executed	The participant turns to the right or left, although they were requested to continue going straight.	Judgement
Opposite turn executed	The participant executes a turn in the opposite direction of what was requested.	Judgement
**Vehicle control**	**The position of the vehicle before the intersection makes it impossible for the participant to complete the request.**	
Driving in the opposite direction	The participant drives in the left lane into oncoming traffic	Judgement
Driving on the shoulder	The participant follows the road, but driving on the shoulder of the road, preventing the participant from accomplishing the turn.	Judgement
Driving off the road	The participant drifts from the road and continues on the grass in another direction.	Judgement
**Crash**	**Simulator displays noise and image of crash.**	
Frequency of crash	Note the frequency of crash and at which intersection they occur	
**Before the Intersection**	**All errors made before the stop line of the intersection**	
**Brakes and speed**		
No use of brakes	The participant attempts to execute the command to turn without the use of the brakes, or does not slow down at all before the turn, or a yellow light.	Attention
Braking	Participant uses brakes too late to execute a turn or avoid an obstacle (results in a sound on the simulator).	Judgement
Exceeding speed limit by 5 km/h to 15 km/h	Exceeding the speed limit by 5 to 15 km/h. Worth one error point on the evaluation scheme.	Judgement
Exceeding speed limit by more than 15 km/h	Exceeding the speed limit by more than 15 km/h. Worth two error points on the evaluation scheme.	Judgement
**Inappropriate stops**	**All stops which are not due to a signal, mark on the ground, or obstacle are inappropriate**	
Excessive preparation	Slowing down right after the command, where the driving speed is less than half the speed limit for a significant distance, but without locking the wheels.	Judgement
Frequency of inappropriate stops after the verbal command	Note the number of inappropriate stops after the verbal command.	Judgement
Exceeding the stop line	In the case where a signal demands a stop, the participant stops the vehicle after already passing the stop line.	Coordination
**Choice of lane**		
Lane deviation	A deviation occurs when at least one of the front wheels is in the neighbouring lane (left or right).	Coordination
Wrong lane	After a command to turn, the participant does not change lanes or stay in the correct lane to be in an adequate position to complete the correct turn (right lane for a right turn, left lane for a left turn).	Judgement
Shoulder lane	The participant drives on the shoulder lane but is still able to complete the correct turn	Perceptual
**Signaling**		
Disobeying signal	Action does not correspond to the signal.	Attention
**Safety**		
Follows too closely	The participant follows the preceding vehicle too closely, putting the driver at a high risk of crash. If the participant’s view did not show the wheels of the vehicle in front of them, the following distance was considered too close.	Judgement
Right of way not given to pedestrian	The participant does not give right of way to the pedestrian.	Attention
**During the intersection**	**Between the stop line of the participant, and that of the drivers going in the opposite direction.**	
**Vehicle control**		
Excessive stopping and anticipation for a turn to the right	Very long stop at the stop line without any apparent reason, even though the light is green. A stop was considered too long when there was no apparent reason why the participant should be waiting any longer to engage in the turn.	Judgement
Very slow execution	Unnecessary braking during the execution of the intersection. A slow execution occurred when there was no apparent reason for participants to use brakes during a turn.	Attention and coordination
Incapable of controlling the vehicle	Loss of control of the vehicle, including inappropriate use of the steering wheel (i.e., participant did not orient the car directly towards the destined road), accelerator (i.e., unnecessary variation of speed during execution), and lane deviations.	Coordination
**Left turn**		
Vehicle is an obstruction in the intersection.	When waiting in the middle of the intersection for space to turn left, the vehicle obstructs the lane going in the other direction.	Judgement
Insufficient time to complete turn	The participant does not have enough space to complete the turn, resulting in a crash.	Attention, perceptual and judgement
Missed turn	Space is available to carry out the left turn, but is not chosen.	Judgement
**Traffic light**		
Disobeying traffic light	Enters the intersection at an inadequate moment and disobeys traffic light.	Attention
**After the intersection**	**The vehicle leaves the intersection marked by a white line on the ground, and up to 30 m afterwards**	
**Speed**		
Speed remains constant	Does not increase speed after a successful turn.	Judgement
Excessive braking	Does not increase speed after a successful turn, and is using the brakes unnecessarily.	Judgement
**Stopping**		
Unnecessary stop after the intersection.	Unnecessary stop once the intersection is completed.	Judgement
**Vehicle orientation**		
Wrong orientation	Unable to return the vehicle to a correct position, even though the initiation was not problematic.	Coordination
Loss of control	The participant orients the vehicle on the shoulder, or in the opposite lane (left), without immediate correction of the situation.	Coordination
